# Histone Variants: Guardians of Genome Integrity

**DOI:** 10.3390/cells9112424

**Published:** 2020-11-05

**Authors:** Juliette Ferrand, Beatrice Rondinelli, Sophie E. Polo

**Affiliations:** Epigenetics & Cell Fate Centre, UMR7216 CNRS, Université de Paris, 75013 Paris, France; juliette.ferrand@univ-paris-diderot.fr (J.F.); beatrice.rondinelli@univ-paris-diderot.fr (B.R.)

**Keywords:** cancer, cell fate, chromatin, chromosome integrity, DNA damage response, DNA repair, genome stability, histone chaperones, histone variants, oncohistones

## Abstract

Chromatin integrity is key for cell homeostasis and for preventing pathological development. Alterations in core chromatin components, histone proteins, recently came into the spotlight through the discovery of their driving role in cancer. Building on these findings, in this review, we discuss how histone variants and their associated chaperones safeguard genome stability and protect against tumorigenesis. Accumulating evidence supports the contribution of histone variants and their chaperones to the maintenance of chromosomal integrity and to various steps of the DNA damage response, including damaged chromatin dynamics, DNA damage repair, and damage-dependent transcription regulation. We present our current knowledge on these topics and review recent advances in deciphering how alterations in histone variant sequence, expression, and deposition into chromatin fuel oncogenic transformation by impacting cell proliferation and cell fate transitions. We also highlight open questions and upcoming challenges in this rapidly growing field.

## 1. Introduction

In cell nuclei, the DNA assembles with histone proteins into chromatin. This highly organized nucleoprotein structure is a source of epigenetic information through modifications affecting the DNA, histone proteins, and variations in chromatin compaction states, which together regulate genome functions by dictating gene expression programs [1]. Chromatin organization also contributes to safeguarding genome integrity, thus protecting cells against tumorigenesis. Indeed, epigenome alterations are frequently observed in cancer [2] and cooperate with genomic alterations to promote tumorigenesis [3]. Several chromatin modifiers were indeed identified as cancer drivers [4] and consistent with this, chromatin modifications play a central role in the maintenance of genome integrity in response to genotoxic stress [5]. Notably, recent findings revealed that not only chromatin modifiers, but also core chromatin components such as histone proteins were altered in cancer [6], which sparked a lot of interest in the oncology field. As we will discuss in this review, it is emerging that histone alterations affect genome integrity at various levels and contribute to oncogenic transformation.

Among histone proteins, core histones H2A, H2B, H3, and H4 assemble into an octamer around which the DNA wraps to form nucleosomes [7]. Nucleosomes are stabilized through binding of linker histones H1, which promotes further chromatin compaction [8]. Importantly, both core and linker histones exist as variants that display similar yet not identical protein sequences and that are encoded by different genes [8,9]. The existence of multiple variants for a given histone type provides an additional layer of chromatin regulation by diversifying nucleosome structure and function. Histone variants affect nucleosome stability and physical properties, and also shape local chromatin environments. They do so by impacting histone post-translational modifications and the recruitment of chromatin-associated proteins, with broad consequences on transcription and cell fate decisions [9,10,11,12]. Two main categories of histone variants can be distinguished on the basis of their expression during the cell cycle. Canonical or replicative histone variants, like H3.1 and H3.2 for H3 variants, are expressed massively during DNA replication in S phase, while replacement variants, including H3.3 and Centromere Protein A (CENPA), are expressed in a replication-independent manner [12]. Both categories of histone variants also differ in their gene organization. In contrast to canonical histone genes that are devoid of introns and grouped into gene clusters, replacement variants are encoded by single genes, which contain introns and therefore can generate functionally distinct splice products. The H2A variant macroH2A1 for instance presents two alternatively spliced isoforms, macroH2A1.1 and macroH2A1.2, which differ in their macrodomain, dictating differential abilities to bind ADP-ribose [13]. In addition to cell cycle-regulated expression, some histone variants are expressed in a tissue-specific manner, restricted to germ cells for instance, as observed for both core and linker histone variants [8,9], while other variants are found only in some species like the hominidae variant H4G [14,15]. Histone variants also display distinct distribution patterns in chromatin, owing to their association with specific histone chaperones [16] and chromatin remodelers [17], which jointly control histone variant deposition and eviction.

Here, we discuss how histone variants and their associated chaperones protect genome stability through the maintenance of chromosomal integrity and by controlling multiple steps of the DNA damage response. This sets the stage for understanding how alterations in histone variant sequence, expression, or deposition into chromatin may have pathological outcomes. Illustrating this point, we review recent progress in elucidating how such alterations contribute to tumorigenesis. Since we aim at describing how histone alterations disrupt genome integrity with a focus on cancer, we mainly present evidence on mammalian histone variants—H2A, H3, and linker histone variants mostly—and refer to other model systems only when relevant. In this review, we do not cover the roles of histone variants in chromatin replication, which are discussed elsewhere [11].

## 2. Histone Variants Safeguard Chromosomal Integrity

**Figure 1:**
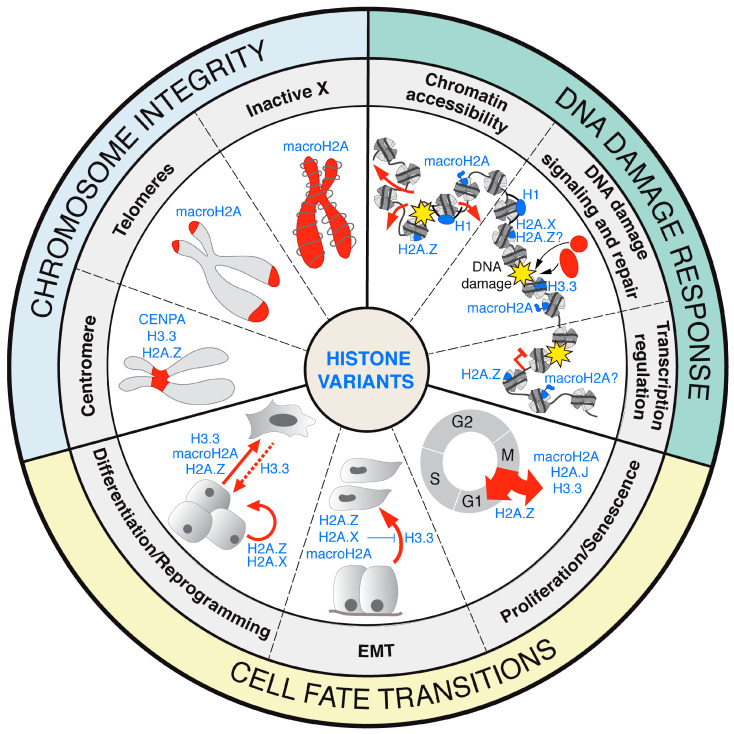
Hallmarks of histone variant contributions to the maintenance of genome integrity and cell identity. Diagram depicting the roles of histone variants in the maintenance of chromosome integrity (blue), the DNA damage response (green), and cell fate transitions (yellow). In each case, key features impacted by histone variants are highlighted in red. EMT: epithelial–mesenchymal transition.

Owing to their ability to define specific chromosomal regions, histone variants are important components in the maintenance of chromosomal stability, at centromeres, telomeres, but also at the level of entire chromosomes such as the inactive X (Figure 1).

### 2.1. Histone Variants Define Centromeric and Pericentromeric Regions Driving Chromosome Segregation

While most of the genomic DNA is packaged with canonical histones, a subset of chromosomal regions is delineated by specific histone variants. One prominent example is the centromeric histone H3 variant CENPA, which is deposited by the Holliday junction recognition protein (HJURP) [18,19] and defines centromere identity and functions [20]. Supporting this, deletion of the *CENPA* gene in mouse embryos impairs the recruitment of centromere components required for kinetochore assembly, resulting in mitotic defects and chromosomal aberrations underlying the lethality of *CENPA* null offspring [21]. In human cells, CENPA ensures proper replication of centromeric repeats, thus preventing centromere breakage and aneuploidy [22]. Overexpression of human CENPA is also a driver for genome instability due to the mislocalization of CENPA-containing nucleosomes on chromosome arms with severe consequences on chromosomal segregation in mitosis [23,24,25,26]. Note that overexpression of CENPA and HJURP have been reported in several cancers ([27] and detailed in Section 4.2), showing that beyond the importance of maintaining CENPA dosage, a tight control of its deposition into chromatin is essential to preserve centromere function, thus safeguarding chromosome integrity.

CENPA is not the only histone variant shaping centromeric chromatin in mammals since nucleosomes containing the H2A.Z variant intersperse with CENPA nucleosomes [28]. Like CENPA, H2A.Z safeguards chromosome segregation from mammals [29] to yeast [30,31]. Furthermore, H2A.Z promotes Heterochromatin protein 1 (HP1) binding to pericentromeric heterochromatin in mouse cells [32] and in Drosophila, where the H2A.Z ortholog H2A.v cooperates with HP1 to stimulate microtubule formation at the kinetochore [33]. This points to the contribution of histone variants at centromeres but also at pericentromeres for governing proper chromosome segregation.

Regarding pericentromeres, the histone variant H3.3 is deposited in pericentric and telomeric heterochromatin by the histone chaperone death domain-associated protein (DAXX) in complex with the chromatin remodeler alpha thalassemia/mental retardation syndrome X-linked (ATRX) [34,35,36]. Consistent with an important function of H3.3 in these heterochromatin domains, mice devoid of H3.3 coding genes display heterochromatin dysfunction impairing chromosome segregation in mitosis and leading to early embryonic lethality [37]. Mutation of H3.3 on lysine 27, a key residue for pericentromeric heterochromatin formation during mouse development, similarly results in mitotic defects and developmental arrest [38].

### 2.2. Histone Variants and Associated Chaperone Complexes Participate in Telomere Maintenance

Besides chromosome segregation that is controlled at the level of centromeres and pericentromeres, the maintenance of telomere length is another fundamental process for chromosomal integrity, which preserves chromosome ends from damage and degradation. Several cancer cells have established a telomerase-independent strategy to elongate telomeric regions named alternative lengthening of telomeres (ALT), which is based on a homologous recombination-mediated DNA replication mechanism [39,40]. Notably, the H3.3-associated remodeler ATRX, and the H3.3 chaperone DAXX to a lesser extent, are frequently mutated in cancer cells and strongly correlate with the ALT phenotype [41]. ATRX overexpression in ALT cells suppresses the ALT phenotype in a DAXX-dependent manner [42]. In addition, ATRX deficiency in human cells induces oncogenic-associated telomere dysfunction [43,44], unraveling the fundamental role of the H3.3 chaperone complex DAXX-ATRX in the maintenance of telomere integrity. It is not yet clear if the function of DAXX-ATRX in ALT is mediated by their ability to incorporate the H3.3 variant at telomeres [34,35]. However, interesting connections between ATRX and macroH2A variants have been unveiled in the context of telomere maintenance. Indeed, ATRX interacts with macroH2A1 and counteracts its association with telomeric chromatin [45,46]. In human cells devoid of ATRX, the histone variant macroH2A1.2 is thus enriched at telomeres and favors homologous recombination-associated ALT pathways [45]. Similarly, in the absence of ATRX, macroH2A1.1 binds to the PARP family enzyme tankyrase 1, preventing tankyrase 1 localization to telomeres, thus promoting aberrant recombination between sister telomeres [43].

### 2.3. MacroH2A Histone Variants Contribute to the Stability of the Inactive X Chromosome

In addition to their roles in telomere maintenance, macroH2A variants also contribute to preserving the integrity of entire chromosomes, as shown for the inactive X [47]. In cells of female mammals, one of the two X chromosomes is silenced during early embryonic development and X chromosome inactivation is then stably maintained during somatic cell divisions [48]. Among other epigenetic features, the inactive X chromosome (Xi) is characterized by an enrichment in macroH2A histone variants [49,50]. Analysis of female viability and mitotic aberrations affecting the Xi revealed that the balance between macroH2A1.1 and macroH2A1.2, generated by alternative splicing of the *H2AFY* transcript, was critical for the stability of the inactive X chromosome in female mammals [47]. This results from opposing activities of these histone variants on DNA repair pathways (detailed in Section 3.2).

Together, these studies illustrate how histone variants, in concert with histone chaperones and remodelers, contribute to the maintenance of chromosomal integrity by delineating functional chromatin domains. Splicing of a histone variant, macroH2A1 also impacts genome stability at the level of an entire chromosome. In addition, histone variants confer a substantial level of plasticity to chromatin [9] which is required for the regulation of all DNA metabolic activities. This is particularly important during the response to DNA damage as discussed in the following section.

## 3. Histone Variants Contribute to the Maintenance of Genome Integrity in Response to DNA Damage

Cells are constantly exposed to genotoxic agents, which trigger a plethora of DNA lesions, including base damage and DNA breaks [51]. For instance, pyrimidine dimers form upon UltraViolet C (UVC) irradiation while ionizing radiation and UVA laser micro-irradiation are commonly used to generate DNA double-strand breaks (DSBs). These lesions are handled by specific repair machineries—excision repair of damaged bases/nucleotides, mismatch repair, break repair—and also activate DNA damage signaling pathways that control cell cycle progression [52]. DSBs in particular can be repaired by three main pathways with different degrees of fidelity: homologous recombination (HR), non-homologous end joining (NHEJ) and alternative end-joining (alt-EJ) [53]. Given that the DNA damage response takes place on a chromatin susbtrate, histone proteins play a central role in this process. While there is no evidence so far that histone variants affect the induction of DNA damage, they impact the DNA damage response at various levels, which we discuss in this section.

### 3.1. Histone Variant Dynamics Control Accessibility and Restoration of Chromatin upon DNA Damage

#### 3.1.1. Fine-Tuning DNA Damage Accessibility through Local and Large-Scale Chromatin Reorganization

**Figure 2:**
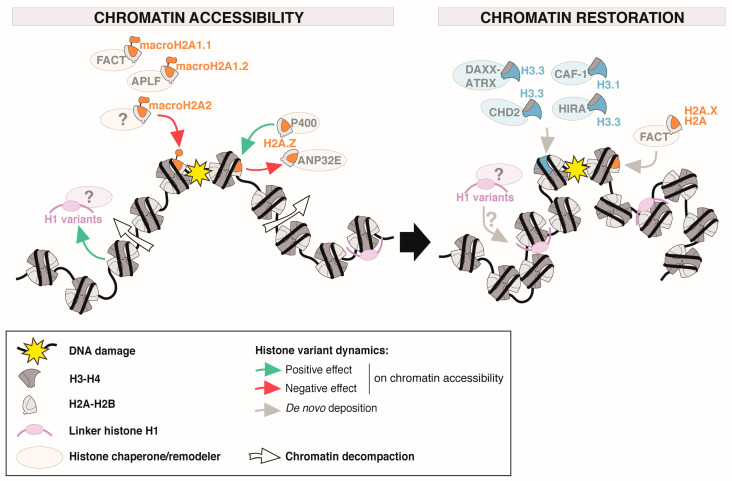
Histone variant roles in damaged chromatin accessibility and restoration. Following DNA damage (yellow star), chromatin accessibility is increased through transient decompaction. The deposition and eviction of specific histone variants in the damaged area positively (green arrows) or negatively (red arrows) contribute to chromatin accessibility. The subsequent restoration of chromatin architecture entails the de novo deposition of histone variants (grey arrows). When known, histone chaperones and remodelers involved in these transactions are indicated. Note that the distinction between histone variant roles in chromatin accessibility and restoration is not that strict: some newly deposited histones also increase chromatin accessibility to repair factors and some histone variants that affect chromatin compaction may persist in chromatin thus contributing to chromatin restoration after damage repair. For simplicity, the responses to different types of DNA lesions are gathered on the same scheme.

DNA repair is accompanied by significant alterations in chromatin structure, which shifts between open and more compact conformations. These changes are tightly controlled in time and space with local and large-scale rearrangements involving histone variant dynamics (Figure 2).

The H2A.Z variant for instance is evicted from chromatin in human cells following UVC irradiation and in response to DNA double-strand breaks (DSBs) in a manner dependent on the histone chaperone acidic nuclear phosphoprotein 32 family member E (ANP32E) [54,55,56]. H2A.Z is also locally and transiently incorporated at DSBs by the chromatin remodeler p400 [57] and by the FBXL10-RNF68-RNF2 ubiquitin ligase complex (FRUCC) [58]. The p400-dependent incorporation of H2A.Z allows subsequent histone acetylation by Tat-interactive protein 60 (TIP60), which promotes chromatin decompaction [57]. It is not yet clear how H2A.Z incorporation and eviction from damaged chromatin are temporally coordinated. Nonetheless, this illustrates the fine-tuning of chromatin decompaction by H2A.Z dynamics. However, this has not been observed in all eukaryotes since the budding yeast ortholog Htz1 is maintained in chromatin upon DSB induction while canonical core histones are partly degraded [59].

The positive effect of H2A.Z on chromatin accessibility is counteracted by macroH2A histone variants, which accumulate in damaged chromatin in mammalian cells [60,61,62,63] and drive chromatin compaction at damage sites, as shown by increased resistance to DNAse I, denser Hoescht staining or contraction of fluorescently-labeled chromatin at laser tracks [60,62,64]. MacroH2A1.1 and macroH2A1.2 variants show differences in their recruitment kinetics to damaged chromatin and also regulate chromatin compaction by distinct mechanisms. Indeed, macroH2A1.1 is rapidly recruited to damaged chromatin in a poly(ADP-ribose) polymerase (PARP)-dependent manner, consistent with the PAR-binding proficiency of its macrodomain [60,61,65]. This coincides with a transient depletion of macroH2A1.2 followed by an ataxaxia telangiectasia mutated (ATM)-dependent re-accumulation of this variant at break sites [62]. The histone chaperones aprataxin-PNK-like factor (APLF) and facilitates chromatin transcription (FACT) promote the accumulation of macroH2A1.1 macrodomain at sites of laser damage [61] and of macroH2A1.2 at sites of replication stress [63], respectively. Altough macroH2A variants accumulate at damage sites, there is no direct evidence so far supporting their bona fide incorporation into nucleosomes at damage sites and some findings even argue against this possibility and support a PAR-dependent association of macroH2A1.1 with damaged chromatin [65]. Mechanistically, macroH2A1 splice isoforms regulate chromatin compaction through distinct pathways: macroH2A1.1 limits chromatin relaxation by inhibiting PARP1 activity [60], while macroH2A1.2 promotes chromatin condensation at DSBs by stimulating the recruitment of the histone methyltransferase PRDM2, which dimethylates histone H3 on lysine 9 [62]. In addition, macroH2A variants promote chromatin compaction via their linker domain [64], which displays structural and functional similarities to histone H1 [66,67]. Indeed, macroH2A linker domain binds DNA at the entry/exit sites of nucleosomes, thus stimulating compaction at the nucleosome and chromatin fiber levels [67,68].

The dynamics of linker histone H1 has also been scrutinized in response to DNA damage with several H1 variants shown to be evicted from chromatin surrounding DSBs [69,70]. H1.2 eviction in particular spans over megabases around the break, highlighting large scale chromatin rearrangements [71]. Note that histone H1 displacement has also been observed at UVC damage sites for H1.0 and H1.4 variants [72]. Given the well established role of linker histones in chromatin compaction [8], it is tempting to speculate that H1 loss from damaged chromatin may contribute to chromatin decompaction and enhanced accessibility to repair factors.

Together, the concerted dynamics of H2A.Z, macroH2A, and H1 variants, which exert opposing activities on chromatin compaction (Figure 2), likely contribute to a fine spatio-temporal regulation of the chromatin state during the repair response.

#### 3.1.2. Chromatin Restoration through De Novo Deposition of Specific H2A and H3 Variants in Damaged Chromatin

In addition to their contribution to chromatin decompaction, histone variants also participate in the restoration of chromatin structure during DNA damage repair (Figure 2). Newly synthesized H2A and H3 variants indeed get deposited at damage sites and may contribute to the final chromatin organization after repair. Such de novo deposition of histones was uncovered for human H3.1 and H3.3 variants in UVC-damaged chromatin by tracking the dynamics of transiently expressed epitope-tagged histones or by exploiting the SNAP-tag technology [73,74]. Note that new histone incorporation in damaged chromatin is not a common feature of H3 variants since the centromeric H3 variant CENPA does not exhibit such behavior [74]. Mechanistically, the H3.1 variant is incorporated by the histone chaperone chromatin assembly factor-1 (CAF-1) coupled to repair synthesis [73], which contrasts with the early deposition of H3.3 promoted by the chaperone histone regulator A (HIRA) at the time of UV damage detection [74]. The de novo incorporation of H3.3 by the chromatin remodeler chromodomain-helicase-DNA-binding protein 2 (CHD2) [75] and by the histone chaperone DAXX in complex with the remodeler ATRX [76] has also been reported at DSB sites in human cells.

Similar to H3 variants, newly synthesized H2A variants including canonical H2A and H2A.X, but not H2A.Z, are deposited in UVC-damaged chromatin by the histone chaperone FACT in human cells [54,77]. This occurs independently of new H3 variant deposition [54].

Importantly, new histones incorporated in UVC-damaged chromatin remain enriched at damage sites for at least 24 h [54,74] and are maintained through cell division, raising the question of their possible impact on epigenetic states and on cell fate after repair. Some of the new histones deposited at DSB sites in contrast might only transiently assemble onto single-stranded DNA generated during DSB resection as shown in yeast cells [78].

After their eviction from damaged chromatin, H1 histone variants re-accumulate as observed on UVA laser tracks [69]. However, the mechanisms underlying the restitution of linker histone positioning are still poorly understood. In particular, it is unclear if they imply the re-incorporation of evicted H1 or the deposition of new molecules, and the molecular players involved in such dynamics are still to be characterized.

Refining our knowledge of chromatin restoration after genotoxic stress will be essential as the contribution of new histone molecules, even if transient, may have a significant impact on epigenetic states. Histone variants deposited into chromatin during DNA repair indeed carry a pattern of post-translational modifications (PTMs), including methylation and acetylation marks, which is distinct from pre-existing histones [79]. They may also constitute a novel substrates for damage responsive PTMs such as phosphorylation and ubiquitylation [80]. This not only influences chromatin structure and functions during repair, but may also have long-lasting effects on the chromatin template. The development of unbiased approaches to track chromatin changes following DNA damage will be critical to evaluate if the pre-damage chromatin state is faithfully re-established and to grasp mechanisms governing the preservation of epigenome integrity.

### 3.2. Histone Variants Control DNA Damage Signaling and DNA Repair Pathway Choice

**Figure 3:**
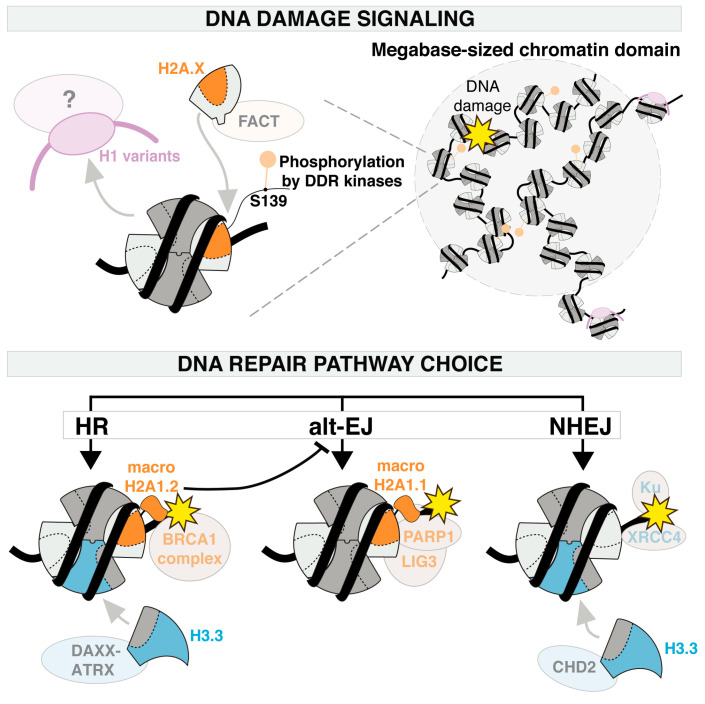
Histone variant roles in DNA damage signaling and repair pathway choice. DNA damage signaling is stimulated by the eviction of H1 histone variants and by the phosphorylation of H2A.X. Prior to phosphorylation, H2A.X is deposited de novo in damaged chromatin by the histone chaperone FACT. These alterations in histone variants span megabase chromatin domains around DNA lesions. H3.3 and macroH2A histone variants drive DSB repair pathway choice by promoting the recruitment of specific repair factors. The H2A.Z variant is not included in this scheme because of conflicting results regarding its impact on DSB repair. DDR: DNA damage response; HR: homologous recombination; alt-EJ: alternative end joining; NHEJ: non-homologous end joining.

Histone variants not only confer a high and controlled level of chromatin plasticity shaping the chromatin substrate for repair, but they also actively contribute to DNA damage responses by stimulating damage signaling and influencing DSB repair pathway choice (Figure 3).

#### 3.2.1. Histone Variants Control DNA Damage Signaling

A key player in the DNA damage response is the histone variant H2A.X, required for checkpoint activation in late interphase after low doses of radiations [81] and for the maintenance of genome stability in mammalian cells [82,83]. Indeed, mice and mouse cells depleted for H2A.X display chromosomal aberrations, defective class switch recombination, sensitivity to radiations, and impaired DSB repair factor recruitment [82,83]. At the molecular level, H2A.X is rapidly phosphorylated by DNA damage responsive kinases, including ATM. This phosphorylation occurs on an evolutionarily conserved carboxy-terminal serine, in position 139 in mammals, leading to the so-called γH2A.X form [84,85,86]. Importantly, this phosphorylation on H2A.X occurs in response to a wide range of DNA damaging agents, supporting the central role of this variant in damage signaling. A fascinating feature of the γH2A.X signal is that it spreads over several megabases surrounding the lesion, as measured after DSB induction, leading to microscopically detectable foci [71,84]. Tridimensional chromatin organization recently emerged as a master regulator of γH2A.X spreading, as put forward in several studies [87,88,89,90]. In particular, super-resolution imaging of γH2A.X foci revealed that topologically associating domains (TADs), which are fundamental modules of higher-order chromatin organization, constrain γH2A.X spreading [88]. Several models, which may not be mutually exclusive, have been proposed to explain the formation of megabase-sized γH2A.X domains: these domains can arise from physical contacts between damaged chromatin fibers within the neighboring chromatin environment [89] and/or through DSB-anchored cohesin-mediated loop extrusion on both sides of the break [90]. The resulting γH2A.X domains constitute a platform for the recruitment of factors governing DSB repair pathway choice like tumor protein P53 binding protein 1 (53BP1) and breast cancer 1 protein (BRCA1) [80,91,92]. Furthermore, H2A.X phosphorylation by ATM counteracts H2A.X ubiquitylation and subsequent proteasomal degradation, thus stabilizing pre-existing H2A.X in damaged chromatin upon DSB formation [93]. In addition, the de novo incorporation of H2A.X by the histone chaperone FACT observed in UV-damaged chromatin may also contribute to potentiate DNA damage signaling [54]. Several complementary mechanisms thus regulate γH2A.X levels in damaged chromatin for an efficient DNA damage response. Even though not characterized with the same level of details as for H2A.X, the H2A.Z variant similarly contributes to DNA damage checkpoint activation in human cells, the depletion of H2A.Z attenuating G2 arrest after damage [57].

Linker histones also control DNA damage signaling but in the opposite way to H2A variants as they prevent hyperactivation of the DNA damage checkpoint and H2A.X phosphorylation in mouse embryonic stem cells [94]. In human cells, the H1.2 variant directly interacts with ATM and suppresses ATM-dependent damage signaling after DSBs [70]. Hence, PARP-dependent displacement of linker histone H1.2 from damaged chromatin promotes efficient damage signaling by ATM.

#### 3.2.2. Histone Variants Stimulate Repair Activities and Guide DSB Repair Pathway Choice

Besides the regulation of DNA damage signaling, histone variants also participate in DSB repair pathway choice. For instance, the early deposition of H3.3 at DSBs stimulated by the remodeler CHD2 promotes the recruitment of non-homologous end joining (NHEJ) repair factors such as KU70-KU80 and X-ray repair cross-complementing protein 4 (XRCC4) [75], while late H3.3 deposition by DAXX-ATRX stimulates DNA repair synthesis during homologous recombination (HR) in human cells [76]. This highlights the importance of H3.3 deposition all along the DSB repair process. H3.3 is also deposited at UV lesions by the histone chaperone HIRA, but HIRA depletion does not affect UV damage repair in human cells [74]. Nevertheless, H3.3 knockout in chicken cells increases cell sensitivity to UV and the H3.3-specific residue serine 31 was identified as key for UV damage tolerance [95]. Along these lines, nematodes lacking H3.3 exhibit sensitivity to oxidative stress [96] and H3.3 and its chaperone HIRA stimulate the expression of stress-responsive genes in plants [97] and fission yeast [98], respectively. Together, this highlights the contribution of H3.3 to genome stability both directly, by stimulating specific steps of DSB repair, and indirectly, through the regulation of transcriptional programs important for genotoxic stress resistance.

Deciphering the impact of H2A.Z on DSB repair is complicated by the fact that this histone variant is found both deposited and evicted from damaged chromatin and conflicting results have emerged regarding the potential involvement of H2A.Z in controlling DSB repair by HR and NHEJ [55,56,57,58,71,99]. However, these studies do not discriminate between H2A.Z.1 and H2A.Z.2 variants, which may have distinct functional outcomes on DSB repair, as exemplified in chicken cells where H2A.Z.2, but not H2A.Z.1, is deposited at DSBs and stimulates HR in response to ionizing radiations [100]. In addition, different activities of H2A.Z in DSB repair may also be mediated, as in transcription, by distinct PTMs on this histone variant, in particular acetylation vs. ubiquitylation [101].

As opposed to H2A.Z, the isoform-specific roles of macroH2A variants in DSB repair pathways are well described in mammalian cells. The concomitant depletion of macroH2A1.1 and macroH2A1.2 increases NHEJ efficiency [65], and each isoform has been shown to stimulate a distinct resection-based DSB repair pathway. Indeed, the ATM-dependent deposition of macroH2A1.2 acts as a positive regulator of HR by promoting the recruitment of BRCA1 to DSBs and to sites of replication stress, thus protecting replication fork integrity [62,63]. MacroH2A1.1 in contrast plays a specific role in alternative end-joining (altEJ) through its ability to bind PARP1 and DNA ligase 3 (LIG3) [47]. Furthermore, macroH2A1.2 counteracts macroH2A1.1 function in alternative end-joining, thus limiting the usage of this mutagenic repair pathway [47]. This antagonism underlines the importance of a tight balance between the two splice isoforms to avoid pathological outcomes. Note that macroH2A1.1 also promotes efficient base excision repair of oxidative damage in human cells by binding and stabilizing PAR chains [102].

Contrary to core histone variants, the role of linker histones in DSB repair pathway choice has not been investigated in mammalian cells and very scarce and contrasting information is reported in other cellular models. The H1R variant has been shown to stimulate HR via Rad54 recruitment in chicken cells [103] while linker histone H1 inhibits HR in yeast and Drosophila [104,105].

How mechanistically histone variants prime the recruitment of repair factors is still elusive. They may locally modify chromatin structure and/or act via specific histone PTMs or reader proteins. For instance, H2A.Z was shown to promote H4K20me2 by recruiting the SUV420H1 methyltransferase, thus controlling replication origin firing [106]. Therefore, H2A.Z eviction from damage sites could impair NHEJ by reducing the 53BP1-recruiting mark H4K20me2, which may also perturb replication timing.

### 3.3. Histone Variants Contribution to Transcriptional Control in Response to DNA Damage

Transcriptional activity at damage sites is rapidly and transiently repressed, which prevents collisions with DNA repair machineries [107,108,109]. However, this needs to be coordinated with chromatin relaxation that usually correlates with an active transcriptional state. The regulation of this delicate balance involves histone modifications [5] but also histone variants and their chaperones.

A link with transcriptional silencing is best established for H2A variants. The FRUCC-dependent ubiquitylation of H2A on lysine 119 stimulates H2A/H2A.Z exchange in damaged chromatin, which leads to transcriptional repression [58]. These findings underline the importance of the balance between H2A variants for the regulation of transcriptional silencing in the damaged area. MacroH2A has been associated with transcriptional repression in several contexts [13]. Consistent with this, the accumulation of macroH2A1.2 at damage sites mediates chromatin compaction and the deposition of the H3K9me2 repressive mark through the recruitment of the methyltransferase PRDM2 [62]. It remains unclear however if this transient heterochromatinization contributes to transcription repression of damaged chromatin.

The histone chaperones HIRA and FACT both promote deposition of newly synthesized histone variants at UV sites and transcription restart in human cells [74,77]. Yet, intriguingly, no connection has been established between their histone chaperone activities and transcription regulation following DNA damage. FACT stimulates transcription restart after UV damage through the recruitment of the transcription-coupled repair factor UV-stimulated scaffold protein A (UVSSA) [110], but the implication of new histone H2A deposition in this process is not established. Furthermore, recent evidence shows that new H3.3 deposition by HIRA in UV damaged chromatin is actually dispensable for transcription restart, highlighting a non-canonical function of the histone chaperone HIRA in this context [111]. Thus, despite their likely contribution to the re-organization of chromatin after repair, the functional importance of newly deposited histones in damaged chromatin still needs to be determined.

In conclusion, histone variants, together with histone PTMs [5], actively contribute to the DNA damage response at multiple levels. Given the central roles of histone variants in controlling chromosomal stability and repair processes, alterations in histone variant dynamics may strongly affect genome stability, thus contributing to tumorigenesis, which we discuss in the next section.

## 4. Histone Variants and Cancer

### 4.1. Point Mutations in Histone Variants (Oncohistones)

**Figure 4:**
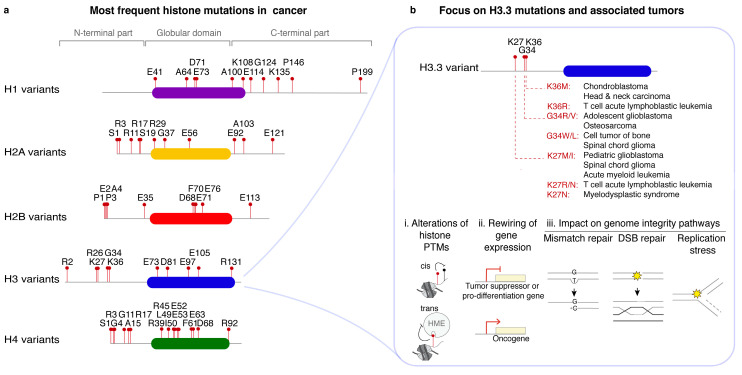
Histone variant point mutations in cancer. (**a**) The ten most frequently mutated residues in each histone family are shown as red lollipops using the single-letter aminoacid code (source [6], Catalogue Of Somatic Mutations In Cancer database [114] (http://cancer.sanger.ac.uk/cosmic). The positions refer to the mature proteins, which lack the initial methionine. Colored bars represent the globular domains of histone proteins. (**b**) *Top*, The most frequent missense mutations in the H3.3 variant are indicated in red, next to the associated tumor types. *Bottom*, Proposed oncogenic mechanisms relying on three types of molecular changes induced by H3.3 mutations. Histone post-translational modifications (PTMs) including methylation and acetylation are affected in cis (on the same histone molecule) or in trans (through inhibition of histone modifying enymes (HME)). Gene expression programs are rewired, leading to the repression of genes promoting differentiation or the expression of oncogenes. Pathways of genome integrity maintenance are also impacted, such as mismatch repair, DNA double-strand break (DSB) repair or the response to replication stress.

In the early 2010s, a series of landmark studies uncovered somatic missense heterozygous mutations affecting H3 histone variants in specific tumor types. Mutant histones were thus put forward as potential oncogenes and hence named ‘oncohistones’ (reviewed in [112]). While genetic alterations have now been identified in all histones in cancer cells [6,113], to date, mutations in histone variants H3.1 and H3.3 are the most characterized and represent the prototype dominant mutations in core chromatin components (Figure 4).

#### 4.1.1. Functional Analysis of H3.1 and H3.3 Oncomutations

Recurrent point mutations in genes encoding the H3.3 histone variant—and less frequently the H3.1 variant—are present in different brain tumors (K27M/I, G34R/V [115,116,117,118,119,120]), in cartilage and bone tumors, soft tissue sarcoma, head and neck cancers (K36M/I and G34W/L [121,122,123]), and in hematological malignancies (K27M/I/N [124,125,126,127,128]). Remarkably, a given amino acid substitution often associates with a specific tumor type, location, and patient age range, by mechanisms that are not completely elucidated. Specific antibodies have been raised against several of these mutations, serving as powerful tools to refine cancer diagnosis. Importantly, the H3.1 and H3.3 point mutations do not affect residues involved in histone chaperone binding and thus do not alter the deposition patterns of these oncohistones into chromatin as shown for the K27M mutation [129,130]. These mutations localize to the N-terminal tails of histones, at or in the vicinity of modifiable residues and affect histone PTMs (methylation and acetylation) through various mechanisms. Mutations can act in cis on the mutated histone molecule [131,132,133] or in trans with genome-wide effects on the histone PTM landscape. The in trans effect of H3 variant mutants derives from their ability to inhibit specific histone modifying enzymes: K27M transient interaction with the H3K27 methyltransferase enhancer of zeste homolog 2 (EZH2) poisons this enzyme and prevents spreading of H3K27me3 from their nucleation sites, thus strongly reducing genome-wide H3K27me3 levels [131,132,134,135,136,137]. K36M traps the H3K36 methyltransferases SET domain containing 2 (SETD2) and nuclear receptor binding SET domain protein 2 (NSD2) and affects H3K36me3 levels [138,139,140], while G34R inhibits H3K9/K36 demethylases of the KDM4 family [141].

**Figure 5:**
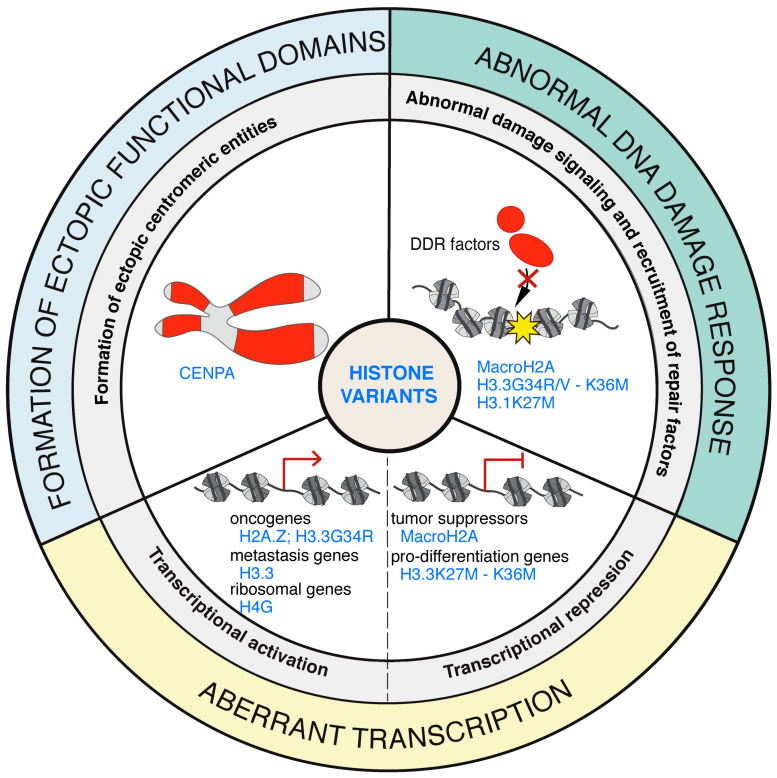
Hallmarks of histone variant dysregulation in cancer. Cellular processes affected by histone variant dysregulation in cancer (highlighted in red) with examples of the associated altered histone variants. DDR: DNA damage response.

Through alteration of histone PTMs, H3 variant oncomutations act as dominant negative, gain-of-function mutations that affect transcriptional programs (Figure 5), generally promoting self-renewal capacities at the expense of cell differentiation programs, with specificities depending on the cellular context and the nature of the mutation [123,131,134,138,142,143,144,145,146]. Through transcriptional dysregulation, H3.3 oncomutations may also affect genome integrity by impacting the number of transcription/replication collisions [147], but studies are still awaited to test this hypothesis.

In addition to their well-established roles in rewiring transcriptional programs impacting cell differentiation, a handful of studies support a contribution of H3 variant oncomutations to altering DNA damage repair pathways (Figure 5). H3.1K27M inhibits NHEJ of DSBs in human fibroblasts [148]. H3.3K36M instead inhibits DSB repair by homologous recombination (HR) in human cells, with consequences on genome stability [138,149]. The proposed underlying mechanism involves loss of H3K36me3, impairing the recruitment of DNA end resection factors [149,150]. The G34R mutation on H3.3 induces replication stress in fission yeast, through a mechanism that involves impaired HR activity [151]. The G34R-dependent inhibition of KDM4B [141] might underlie HR inhibition through aberrant H3K9me3 levels [152]. In addition, mutations affecting the G34 residue also impair interaction of the neighboring H3K36me3 modification with the mismatch repair protein MSH6, resulting in genome instability [153]. Altogether, these studies uncover that H3 variant oncomutations may disrupt the response of cells to DNA damage, ultimately impacting genome stability. One important future challenge will be to determine to which extent disruption of repair pathways in H3 mutant cells contribute to oncogenesis and if alterations in DNA repair pathways can be exploited for patient management and offer new therapeutic opportunities.

Noteworthy, even though H3.1 and H3.3 variants can bear identical amino acid substitutions in cancer, there are significant differences between these mutated histone variants. For instance, H3.1K27M and H3.3K27M are differentially distributed in chromatin and H3.1K27M results in stronger reduction of H3K27me3 levels [130]. H3.1K27M and H3.3K27M also promote distinct oncogenic transcriptional programs in diffuse intrinsic pontine gliomas (DIPGs) [120,154] involving variant-specific patterns of active enhancers [143]. Accordingly, H3.1K27M and H3.3K27M DIPGs are histologically and clinically distinct: H3.3K27M tumors have a later onset but are more aggressive and more resistant to radiotherapy than H3.1K27M tumors [120], which is driven by the co-occurrence of the H3.3 mutation with p53 inactivation [155]. It will be interesting to assess whether H3.1K27M and H3.3K27M oncomutations also differently affect repair of DNA damage.

Beyond TP53, H3.3 mutations were shown to co-occur with other genetic alterations, such as amplification of platelet-derived growth factor receptor α (PDGFRα) or mutations in the DAXX-ATRX chaperone complex [116,156,157]. Contrary to DAXX-ATRX, no mutations in the HIRA chaperone complex were identified in H3.3 mutant cells, suggesting that the H3.3 chaperone HIRA likely mediates the deposition of these oncohistones into chromatin. H3.3K27M was shown to synergize with TP53 loss and PDGFRα activation to drive neoplastic transformation [144,146,158]. Further investigations will help dissect the functional cooperation of H3 variant mutations with co-occurring genetic alterations in promoting tumor initiation and progression.

#### 4.1.2. Mutations in other Core Histones

Point mutations in genes encoding for H2A and H2B variants were initially identified in uterine [159], bladder and head and neck cancers [160]: R4H, K16T, E57Q in H2A; G27A, E36G, M63K, E76K in H2B. These mutations were shown to alter genome-wide chromatin accessibility and gene expression, possibly by affecting PTMs, because lysine and arginine are modifiable residues, or histone–histone interactions [159,160], as it is the case for H2B E76K [161]. The spectrum of cancer-associated histone mutations recently expanded to all core histones and to a vast array of tumor types [6]. The identified mutations not only map to the N-terminal tail but also to other structurally and functionally critical regions in globular domains such as the acidic patch. Structure-function analyses indicate that the mutations are likely to affect nucleosome stability, higher-order folding of chromatin, to mimic histone modifications or even to generate neo-substrates for histone modifying enzymes [6]. These works consolidate histone proteins as key components of epigenetic dysregulation in cancer. Future studies should aim at functionally characterizing these mutations and unravel their relevance in driving tumorigenesis. Given the crucial contribution of histone variants to genome integrity maintenance (as seen in Section 2 and Section 3), it is temping to speculate that at least some histone mutations may fuel tumorigenesis by contributing to the induction of genome instability.

#### 4.1.3. Mutations in Linker Histones

Recurrent mutations of the linker histone genes HIST1H1 B–E were identified in hematological malignancies, especially lymphomas, and in solid tumors (reviewed in [113]). Their recurrent, missense and heterozygous nature may suggest a driver role in tumorigenesis, but more studies are needed to dissect the underlying mechanisms, which may involve impaired protein-DNA or protein–protein interactions, or alterations of H1 PTMs such as lysine methylation. H1 variant oncomutations may also hinder the function of linker histones in the response to DNA damage (detailed in Section 3).

### 4.2. Misexpression and Misincorporation of Histone Variants into Chromatin

**Figure 6:**
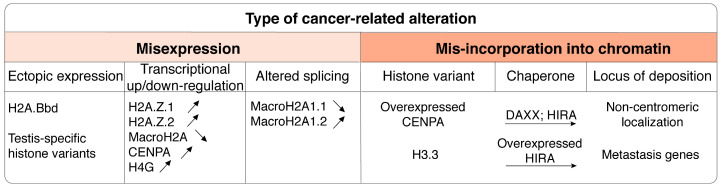
Alterations of histone variants in cancer. Cancer-associated histone variant alterations include, in addition to point mutations (see Figure 4), different types of misexpression (up and down arrows indicate overexpression and downregulation, respectively) and incorporation in chromatin at ectopic loci, mediated by specific histone chaperones.

Accumulating evidence shows that histone variants are misexpressed in cancer cells. Misexpression includes up or downregulation, ectopic expression, and splicing alterations. In addition, the incorporation patterns of histone variants into chromatin can also be altered due to dysregulation of their deposition/eviction machineries (Figure 6).

#### 4.2.1. Cancer-Associated Alterations in H2A Variants

Among the members of this vast family of histone variants, macroH2A and H2A.X are generally considered as tumor suppressors, while H2A.Z acts as an oncogene [162]. The three macroH2A variants—macroH2A1.1, 1.2, and macroH2A2—are commonly downregulated in malignant, metastatic, and recurrent tumors, including melanoma, bladder, lung, and gastro-intestinal cancers (reviewed in [162]). This pattern of expression is associated with tumor suppressive roles of macroH2A variants in controlling cell proliferation and self-renewal programs through transcriptional regulation. For example, macroH2A limits the proliferative potential of melanoma and breast cancer cells by hindering the expression of cyclin-dependent kinase 8 (CDK8) [163,164]. The cell cycle inhibitor p16 is another target gene of macroH2A1-dependent silencing in cancer cell lines [165] but we still ignore if this contributes to regulating proliferation in primary tumors. Furthermore, the diminished expression of macroH2A in advanced cancer fosters self-renewal traits and transcriptional programs, as shown in hepatocellular carcinoma and bladder cancer [166]. In addition to misexpression, the splicing pattern of macroH2A1 is also altered in cancer cells. This is relevant since macroH2A1 splice isoforms entail different tumor suppressive functions. Biased splicing towards the macroH2A1.2 isoform at the expense of macroH2A1.1 is reported in several cancer types [167,168,169,170], through downregulation of the QKI splicing factor [168], or through the activity of the DDX5 and DDX17 RNA helicases [167]. The reduced levels of macroH2A1.1 correlate with the proliferation rate of breast and lung cancers [171] and predict patient survival in colon cancer [169]. On the other hand, a macroH2A1.2-specific tumor suppressive function was identified in osteoclastogenesis, a metastasis-dependent bone resorption process [172,173]. MacroH2A1.2 indeed cooperates with epigenetic regulators to inhibit the expression of osteoclastogenic-promoting factors secreted by cancer cells.

Contrasting with macroH2A, overexpression of H2A.Z variants has been observed in several cancers including melanoma, bladder, prostate, and breast cancers, where it correlates with poor prognosis (reviewed in [162]). The oncogenicity of H2A.Z overexpression relates to H2A.Z function in transcriptional regulation, with increased incorporation of H2A.Z at transcription start sites of genes promoting cell proliferation [174] and at enhancers involved in hormone-dependent signaling in cancer cells [175,176]. Variant-specific functions of H2A.Z.1 and H2A.Z.2 in cancer are mediated by their incorporation at specific gene regulatory regions. In melanoma, H2A.Z.2 is incorporated at E2F-target, cell cycle-promoting genes and mediates their overexpression, in cooperation with bromodomain-containing protein 2 (BRD2) [177]. Loss-of-function of this oncogenic axis renders melanoma cells sensitive to chemotherapeutic drugs, uncovering an interesting Achille’s heel of H2A.Z.2 overexpressing cells [177]. On the other hand, overexpressed H2A.Z.1 controls the expression of key regulators of the cell cycle and of the epithelial–mesenchymal (EMT) transition and is associated with poor prognosis in liver cancer [178]. In addition to H2A.Z variants themselves, overexpression of H2A.Z deposition/eviction machineries is also reported in cancer cells and of relevance for cancer progression and prognosis [179,180,181,182,183,184,185].

The gene encoding the H2A.X variant maps to a chromosomal region, the 11q chromosomal arm, which is frequently mutated and deleted in cancer but a formal demonstration that alterations in this gene are responsible for the transformation of human cells is lacking. Nevertheless, loss-of-function studies of H2A.X in several model systems support its role as a caretaker since H2A.X limits the occurrence of genome instability (discussed in Section 3). In particular, loss of H2A.X in mice dramatically increases the frequency of lymphomagenesis in a dosage-dependent manner, with tumors featuring gross chromosomal translocations [186,187].

The less characterized H2A Barr body-deficient variant (H2A.Bbd) is ectopically expressed in Hodgkin’s lymphoma cells and is considered a cancer-associated antigen and a possible target of immunotherapy [188]. Ectopic expression of H2A.Bbd in mouse embryonic stem cells leads to its incorporation into chromatin at DNA damage sites and its expression confers increased damage sensitivity [189]. More studies are awaited to clarify the relevance of these biological roles in disease and specifically in cancer.

#### 4.2.2. Cancer-Associated Alterations in H3 and H4 Histone Variants

The centromeric H3 variant CENPA [190,191,192,193,194] and its cognate chaperone HJURP [195,196,197,198,199,200,201] are overexpressed, usually in a mutually exclusive fashion, in several cancer types including lung, colon, prostate, breast, and brain tumors, where their overexpression correlates with poor prognosis. CENPA epigenetically marks centromeres (reviewed in [20]) but gets mis-incorporated at chromosomal arms by the histone chaperones DAXX and HIRA when overexpressed [24,25,26]. This creates ectopic centromeric entities contributing to chromosomal instability and aneuploidy [23] (Figure 5). H3 and H4 total levels are not affected by CENP-A or HJURP overexpression [24]. These findings build a strong connection between CENPA function in chromosome segregation and its role in tumorigenesis. CENPA overexpression also renders cells more tolerant to DNA damage [24], which may strongly compromise genome integrity even if the underlying mechanism is currently not clear. A possible role of p53 in conjunction with CENPA in oncogenesis is suggested by recent studies showing that the p53 status is a key determinant of the impact of HJURP and CENPA overexpression on cell identity, genome stability, and response to therapy [202,203]. At the molecular level, CENPA forms homotypic nucleosomes at centromeres [204], while heterotypic nucleosomes containing both CENPA and H3.3 are observed in CENPA-overexpressing cells [24]. These heterotypic nucleosomal structures are extremely stable [205], and enriched at genomic sites of high histone turnover, such as gene regulatory elements [24,25]. Whether and how this incorporation contributes to the oncogenesis of the heterotypic H3.3-CENPA nucleosomes still awaits further characterization. Nevertheless, these works raise the possibility that overexpressed CENPA may contribute to oncogenesis not only via chromosomal instability but also by perturbing transcriptional programs. Supporting this idea, recent data show that CENPA is required for the transcription of genes involved in cell proliferation and consistently, CENPA overexpression drives proliferation in prostate cancer cells [194].

CENPA is not the only H3 variant whose levels and deposition pattern are altered in cancer. The H3.1/H3.3 deposition balance is also affected through dysregulation of CAF-1 and HIRA chaperone expression. Indeed, the replicative H3 variant-specific chaperone CAF-1 is overexpressed in tumors compared to normal tissues as first observed in breast cancer, where CAF-1 expression serves as a proliferation and prognostic marker [206]. During metastasis induction, in contrast, CAF-1 expression is downregulated while the H3.3-specific chaperone HIRA is upregulated. This ultimately favors the ectopic incorporation of H3.3 in place of H3.1, which fuels a pro-metastatic transcriptional program [207]. Note that in these studies, H3 chaperone levels rather than H3 variant expression are affected in cancer cells, thus impacting H3 variant deposition patterns. Nevertheless, one report indicates that H3.3 levels are decreased in adult glioblastoma cells [208], which contrasts with H3.3 gain-of-function mutations observed in children and young adult glioblastoma (detailed in the previous section).

The newly discovered hominidae-specific histone H4 variant H4G is overexpressed in breast cancer cells, in a tumor stage-dependent manner. Oncogenic features of H4G rely on its ability to stimulate ribosomal RNA synthesis, which ultimately promotes protein production and cell growth [14,15].

#### 4.2.3. Linker and Tissue-Specific Histone Variants: Few Hints for a Widespread Role in Cancer

H1 histone variants undergo a highly heterogenous dysregulation in cancer, with differences depending on the type of histone variant, the tissue, and tumor stage (reviewed in [113]). Generally, the expression of replication-dependent variants such as H1.5 correlates with cancer progression [209,210], while replication-independent variants such as H1.0 show reduced expression in advanced cancers [211,212]. The pattern of H1 variant expression could thus be a resourceful biomarker of tumor malignancy. It is unclear if the increased level of replication-associated histones is a cause or a consequence of increased cell proliferation in advanced tumors. Actually, the functional relevance of H1 variant dysregulation in cancer is still a subject of investigation. Mechanistically, we know that H1.0 contributes to silencing the expression of oncogene effectors [213]. Other points of interest are the reported interactions of H1 variants with known tumor suppressors, PTEN [214] and p53 [215,216], and the contribution of H1 variants to DNA damage responses (detailed in Section 3), through which they may exert their pro- or anti-tumorigenic effects.

Several germ cell-specific core and linker histone variants display aberrant expression in tumors [217] and thus belong to the so-called family of cancer/testis antigens [218], but how they contribute to oncogenesis is still elusive.

### 4.3. Importance of Histone Variant Post-Translational Modifications in Cancer Development and Monitoring

PTMs of histone variants modulate their functions and contribute to their oncogenic roles. Therefore, the detection of histone variant PTMs can be used as a clinical biomarker. For example, phosphorylation of H3.3 on serine 31 expands from pericentromeric regions to whole chromosomes in a checkpoint kinase 1 (CHK1)-dependent manner and contributes to the maintenance of genome integrity in ALT cancer cells [219]. Aberrant PTMs on CENPA, including methylation and phosphorylation, impair CENPA deposition at centromeres and the recruitment of kinetochore components, leading to chromosome missegregation, which fuels tumor formation (reviewed in [220]). PTMs on H2A variants similarly contribute to oncogenesis. H2A.Z acetylation (H2A.Zac) for instance, which marks the promoters of highly expressed genes, is depleted from tumor suppressor gene promoters and redistributed to the promoters of active oncogenes and to androgen-responsive neo-enhancers in prostate cancer [175,221]. H2A.Zac redistribution thus results in ectopic gene expression and H2A.Zac levels correlate with poor cancer prognosis [175]. Methylation is another PTM on the H2A.Z variant driving oncogenesis. H2A.Z.1 methylation by SET and MYND domain containing 3 (SMYD3) indeed prevents the removal of this histone variant from chromatin by ANP32E and stimulates the expression of growth-associated genes including cyclin A1, thus promoting breast cancer cell proliferation [222]. Last but not least, the damage-responsive phosphorylation of H2A.X on serine 139 (γH2A.X, described in Section 3) is a valuable diagnostic and prognostic marker in multiple solid tumors [223]. γH2A.X levels can even be measured in peripheral blood, where they reflect the susceptibility to damage and the efficiency of damage signaling in a given organism, thus serving as a non-invasive biomarker of cancer risk [224,225,226]. Furthermore, the ability of cancer cells to detect and signal DNA damage is a measure of efficient treatment response. Therefore, γH2A.X is used in several clinical studies to monitor the response to radio- and chemotherapies [227,228]. Histone variant PTMs thus appear as an important additional layer of regulation in tumorigenesis, of clinical relevance for cancer diagnosis and prognosis.

### 4.4. Histone Variants Regulate Cell Proliferation and Cell Fate Transitions Impacting Tumorigenesis

Histone variants contribute to balancing progression and exit of the cell cycle, and also cell differentiation and self-renewal capacities (Figure 1). As a consequence, their misexpression, misincorporation, or mutation hijack these tumor suppressive functions and contribute to the acquisition of cancerous cell traits such as uncontrolled proliferation, stemness, or aberrant migratory phenotype.

#### 4.4.1. Control of Cell Cycle Progression and Exit

Several studies support a role for histone variants in regulating cancer cell proliferation by controlling the expression of cell cycle regulators. Indeed, H2A.Z.1 and H2A.Z.2 variants induce the expression of proliferation-promoting genes, either directly through H2A.Z deposition at the promoter of these genes or indirectly by enhancing hormone-dependent signaling [99,174,177,178,229]. MacroH2A variants in contrast limit cell proliferation by silencing CDK8 in melanoma and breast cancer [163,164]. Note that this mechanism cannot be generalized since macroH2A1.1 expression restricts cell proliferation independently of CDK8 in lung cancer cells [168].

Senescence is a permanent cell cycle arrest, accompanied by the production of senesence-promoting cytokines, referred to as senescence-associated secretory phenotype (SASP) [230]. MacroH2A1.1 exerts tumor suppressive functions by binding to SASP genes and stimulating their expression, thus triggering senescence in a paracrine fashion [231]. Similarly, the poorly characterized H2A.J variant accumulates in senescent cells and promotes SASP gene expression [232]. MacroH2A1.2 instead protects against replication stress-induced senescence [63] and H2A.Z suppresses senescence by repressing the p53-p21 cell cycle regulatory pathway [233]. The H3.3 variant and its specific chaperone HIRA promote a senescence-associated transcriptional program upon oncogene stimulation, enforcing tumor suppression [234,235], while the H3.3-associated remodeler ATRX is required for drug-induced senesence [236]. Finally, several histone variants and associated chaperone complexes counteract replicative senescence through their contribution to telomere maintenance via the ALT pathway (discussed in Section 2). Collectively, these studies reveal multifaceted roles of histone variants in controlling cell cycle progression and exit to senescence, with important implications for tumorigenesis.

#### 4.4.2. Impact on Cell Differentiation, Self-Renewal, and Reprogramming

Several histone variants, including macroH2A, H2A.Z, and H3.3, strongly impact the balance between cell differentiation and self-renewal by regulating the expression of pluripotency and developmental genes. The deposition of H3.3 at the promoters of developmental genes indeed drives the differentiation of embryonic stem cells and regulates neuronal differentiation programs [237,238,239]. Similarly, macroH2A variants stimulate the differentiation of pluripotent cells in mammals [240,241] and, in agreement with these findings, macroH2A loss enhances cell stemness in liver and bladder cancer [166,242]. The case of H2A.Z is more complex, this histone variant promoting both self-renewal and differentiation of mouse embryonic stem cells [243,244]. The phosphorylation of H2A.X on serine 139 is also important for the maintenance of stem cell self-renewal capacities [245]. Indeed, H2A.X-null mouse embryonic stem cells show impaired self-renewal, which is restored by expressing wild-type but not S139A mutant H2A.X.

In addition, histone variants and their chaperones control the ability of differentiated cells to reprogram to a pluri/totipotent state. H3.3 deposition stimulates reprogramming upon nuclear transfer in Xenopus and mouse oocytes [246,247] while the replicative H3 variant-specific chaperone CAF-1 represents a barrier to reprogramming in mammalian cells [248,249]. Several groups have also shown that macroH2A variants, mostly macroH2A2, hinder reprogramming by preventing the activation of pluripotency genes [250,251,252,253]. In contrast, the expression of oocyte-specific H2A and H2B variants facilitates the reprogramming of fibroblasts [254]. The effects of other histone variants, including H2A.Z and testis-specific linker histone variants, on reprogramming are still uncharacterized. Nevertheless, these findings illustrate tight links between multiple histone variants and the control of cell fate transitions, which is of pivotal importance during tumorigenesis when cells switch towards a stem-like state.

#### 4.4.3. Regulation of the Epithelial–Mesenchymal Transition (EMT)

Among key cell fate transitions, the epithelial–mesenchymal transition (EMT) is a developmental process that can also drive tumor invasion and metastasis [255]. Several histone variants prevent the occurrence of EMT: H2A.X, by transcriptional repression of mesenchymal-promoting genes [256] and so does the macroH2A1.1 isoform, in a PAR-dependent manner [257]. Always through transcriptional regulation, H2A.Z can either promote [178] or prevent EMT [258], depending on the cancer context, pointing to a complex regulatory network contributing to EMT beyond histone variant incorporation. Dysregulation of histone variant deposition patterns and mutations in histone variants also promote the EMT. H2A and H2B mutations found in uterine and ovarian cancers, for instance, induce the expression of EMT markers, stimulating cell migration and invasion [159]. Unbalanced expression of the two chaperone complexes CAF-1 and HIRA promotes EMT through ectopic incorporation of H3.3 at metastasis-inducing genes [207]. Together, these studies highlight how histone variants control tumor invasion through EMT regulation.

## 5. Conclusions and Perspectives

The histone variant field is rapidly evolving with new variants recently characterized and a growing number of histone oncomutations identified, opening new horizons in cancer research. Among their various roles in controlling genome functions, histone variants contribute to the maintenance of genome integrity at multiple levels, by regulating chromosomal stability, the chromatin response to DNA damage, but also transcriptional programs and cell fate transitions (Figure 1). In contrast to damage-induced histone PTMs, which are removed concomitantly with termination of DNA damage signaling and repair, histone variants incorporated into damaged chromatin may persist after repair and thus have long-term effects on the epigenetic landscape. Future studies will examine the existence of such damage scars and their consequences on epigenetic states and cell fate.

Mutations or misexpression of histone variants and their chaperones are common in cancer and lead to aberrant gene expression and impaired genome integrity. Furthermore, histone variant alterations often correlate with cancer progression, highlighting a widespread, potential diagnostic and prognostic relevance. For many variants and chaperones, however, it is currently unclear whether misexpression is a bystander effect or if it has a driving role in the oncogenic process. Dissecting their contribution to tumorigenesis through the alteration of epigenetic marks and subsequent rewiring of transcriptional programs, but also through alterations in genome integrity, will likely offer multiple therapeutic angles. In addition to using drugs targeting epigenetic marks (epidrugs), it will indeed be possible to exploit vulnerabilities of cancer cells to specific genotoxic drugs, alone or in combination with epidrugs. Furthermore, neoantigens derived from oncohistones may provide targets for immunotherapy. As regards to mutations in histone variant genes, we expect, in the years to come, a burst of studies aimed at characterizing their oncogenicity and their functional consequences at the molecular and cellular levels. Future work should also help deciphering possible crosstalks between histone variants in the regulation of genome integrity. Finally, a thorough analysis of histone variant alterations in individual tumor cells will be instrumental for assessing the heterogeneity of chromatin states in cancer.
